# Exploring Facilitators and Barriers to Physical Activity for Families of Rural Preschoolers Participating in a Motor Skill Program

**DOI:** 10.3390/children11030362

**Published:** 2024-03-19

**Authors:** Amanda Campbell, Jill Lassiter, Michael Ertel, Andrea R. Taliaferro, Mackenzie L. Walker, Ali S. Brian

**Affiliations:** 1 Department of Health and Human Sciences, Bridgewater College, Bridgewater, VA 22812, USA; acampbel@bridgewater.edu; 2 Department of Health Sciences, James Madison University, Harrisonburg, VA 22801, USA; lassitjw@jmu.edu; 3 Department of Educational and Developmental Science, University of South Carolina, Columbia, SC 29208, USA; ertel@email.sc.edu (M.E.); taliafa@mailbox.sc.edu (A.R.T.); mlwalker@email.sc.edu (M.L.W.)

**Keywords:** early childhood, movement skill, caregivers, parents, intervention, social support, access, resources, motor development, children

## Abstract

While schools provide one opportunity to encourage physical activity, caregivers play an exceedingly important role in creating an environment conducive to preschool children’s physical activity. Yet, little is known regarding the perceptions of caregivers, important choice agents for young children’s physical activity behavior after participating in a motor skill program. The purpose of this study was to examine caregivers’ perceptions of facilitators and barriers to children’s physical activity at home among rural, low-income families who participated in a school-based early childhood physical activity program, SKIPping with PALS, designed to increase physical activity and improve motor development. Eleven caregivers consented to participate in a semi-structured interview regarding their perceptions of physical activity and their experience after six months of participation in the program. An inductive, naturalistic evaluation approach was utilized for qualitative data analysis, following the six recursive phases of thematic analysis. A review of the interview transcripts revealed that all caregivers valued physical activity and encouraged their children to be active. Four major facilitators, four major barriers, and an overarching theme of parental support for childhood physical activity were identified. These factors are largely circumstantial and attitudinal and, thus, are difficult to modify but are important to be cognizant of when designing interventions.

## 1. Introduction

Physical activity participation in early childhood is a powerful deterrent of hypokinetic disease [1,2,3] supports social-emotional development [4], predicts school readiness [5], and promotes the learning of gross motor skills, the building blocks of lifespan movement/physical literacy [6,7,8,9,10]. In contrast, sedentary behavior in early childhood predicts early mortality [11] and developmental difficulties that cascade across numerous domains (e.g., psycho-social, physical, social-emotional) [12,13]. Many young children are adapting to the current environment that encourages more screen time and technology use and less active play, which results in an imbalance in health-enhancing physical activity behaviors, compared to obesogenic behavior [14,15,16]. These more sedentary lifestyle behaviors in the early years predict a developmental trajectory towards inactivity, developmental delays, poor school readiness, and early mortality [15,17,18]. Children 3–4 years of age should be physically active for at least 180 min per day (24 h), of which at least 60 min should be at a moderate to vigorous level [19,20]. Adherence to the WHO guidelines on physical activity varies by country but appears to be consistently low. For example, in Canada, where the original guidelines were developed, only around 13% of 3–4-year-olds are meeting the daily physical activity guidelines [21] and in Japan, about 21% of preschool children (aged 3–4 years) met the guidelines [22]. Given the worldwide issue of adherence to physical activity guidelines for preschool age children, identifying the factors that contribute to early childhood physical inactivity and motor development delay is an important step to mitigate the negative consequences associated with lifetime physical inactivity.

Unfortunately, young children from rural settings in the USA face health disparities when compared to their non-rural counterparts, placing them at a heightened risk for the above mentioned negative developmental trajectories (including gross motor skill difficulties) [23,24] and inciting rural living as a social determinant of health [25,26,27]. Reasons for these health disparities may vary; however, disparities refer to a difference in treatment or level that may be perceived as unfair [28]. According to Sallis et al. [29] and their Ecological Model of Active Living, there are many facets in the environment that impact physical activity, ranging from personal- to systems-level aspects, which transcend across four core principles. These four core principles state that (1) there are multiple levels of factors that influence health behaviors, (2) these influences interact across levels, (3) multi-level interventions should be most effective in changing behavior, and (4) ecological models are most powerful when they are behavior specific. Thus, those who reside in rural areas in the USA may have exacerbated challenges to those areas deemed requisite by Sallis et al. [29]. For example, those in US rural communities are often further away from neighbors and community resources, thus geographically based health disparities can be attributed to a lack of access to social supports, lack of transportation to parks, playgrounds, sporting clubs/teams/facilities, and other community- and health-promoting resources [30,31]. Additionally, US rural youth are much less active than their urban counterparts and are 25% more likely to be obese than urban children [32,33].

Another social determinant of health for children living in rural areas is poverty. Poverty exacerbates health disparities among children in rural areas, posing significant obstacles to their overall well-being [34]. Limited financial resources often translate into inadequate access to nutritious food, quality healthcare, and educational opportunities [34]. Families grappling with economic challenges may find it difficult to afford nutritious meals, leading to potential nutritional deficiencies and developmental issues in children. Additionally, the scarcity of affordable healthcare services in rural regions in the USA can result in delayed or insufficient medical attention, hindering preventive care, and exacerbating health conditions [35]. The intersection of poverty and limited access to quality education further perpetuates a cycle of disadvantage, limiting the opportunities for these children to achieve optimal health outcomes [36]. Addressing the complex interplay between poverty and health in US rural communities is crucial for fostering a foundation of well-being for children and promoting a more equitable and healthier future.

A common approach to combating the negative outcomes associated with barriers and obesogenic behavior for children in rural environments is virtual programming [37]. Virtual programming exploded during the pandemic; however, for those in rural environments, a lack of internet access remains a health disparity [38], negating the potential support gained from virtual programming. A plausible option for those experiencing health disparities based upon residing in a rural environment is a hybrid (face-to-face/virtual) approach that involves parents/guardians and teaches them to utilize the abundant space so common among rural households [39]. Additionally, certain factors can facilitate physical activity among children. These factors include communication/collaboration between teachers, families, students, and stakeholders, programming implementation climate, staffing capacity/continuity, physical activity opportunities throughout the school day, and staffing adaptability [40].

Additionally, school-based programming with direct parental involvement has the potential to improve children’s weight status, physical activity, and sedentary behavior [41]. Efficacy regarding parental involvement with school-based programming is not surprising given the strength of past evidence supporting this dyad [22,42,43,44,45,46]. To demonstrate parental influence, the relationship between maternal and child physical activity shows that for every step a mother took, their child took 1.2 steps [47]. Furthermore, parents are the choice agents for their young children. The strength of the association of parental influence decreases across time, making it critical to involve parents and primary caregivers both in behavior and developmental facets of physical activity at an early age [48].

Recognizing previously known barriers and facilitators for children in rural communities, Brian et al. [1] developed and implemented SKIPping with PALS to mitigate obesogenic behaviors through education and practice with direct parental involvement (e.g., addressing the behavioral change across multiple levels, as recommended by Sallis et al. [29]). The direct results of SKIPping with PALS on the objective measures of physical activity were powerful, with children seeing increases in their behavior as great as 1500 steps per day per week [1]. These results are in line with the previous literature that demonstrates children’s motor skills were positively related to their engagement in vigorous physical activity [15]. However, it is important to understand exactly why parents and children achieved such a great benefit from SKIPping with PALS when compared to the previous early childhood physical activity intervention literature, which tends to only see a 10-min-per-day increase in physical activity behaviors when not directly involving parents [49]. Therefore, the purpose of this study was to examine the facilitators and barriers to childhood physical activity as perceived by parents living in low socioeconomic status and rural areas.

## 2. Materials and Method

### 2.1. Participants

All families of enrolled children at the early childhood center were invited to participate in SKIPping with PALS. Next, all caregivers (≈120 families) who opted into the SKIPping with PALS program were invited to participate in interviews about their experiences. Eleven caregivers (ages ranging from 25 to 63 years) volunteered and consented to participate in a semi-structured interview regarding their perceptions of physical activity, their children’s engagement with physical activity, and the SKIPping with PALS program (see Table 1 for demographics on caregivers and their children). All participants had an annual income below USD 30,000. The median income for a family of four in this location within the United States is USD 58,234 [50]. In the location of the United States where this study was conducted, the federal poverty level for a family of four is USD 30,000, which is used by the Department of Health and Human Services to qualify a family for federal nutrition and healthcare assistance programs [45]. Fourteen percent of families in this state live below the poverty line [50]. This project was approved by the participating institute’s Institutional Review Board, protocol number Pro00089699. All participants provided written informed consent.

### 2.2. Intervention

SKIPping with PALS is a school-based early childhood program designed to increase physical activity and improve motor development among young children, described in detail by Brian et al. [1]. In summary, once per week, parents were invited to attend brief educational sessions at the early childhood center, followed by guided physical activity time with their children. Parents also received take-home equipment bags and access to online resources to help facilitate activity with their children at home. The program demonstrated effectiveness at increasing children’s physical activity levels and gross motor skills, with a direct linear relationship between the number of sessions a parent attended and the outcome measures [1].

### 2.3. Data Collection

Interviews took place in March 2020 after parents had the opportunity to attend six in-person SKIPping with PALS sessions, just prior to the COVID-19 lockdown. Interviews were conducted over the phone by a member of the research team (MW). Interviews were transcribed by two research team members (AC and JL) and checking of transcripts was performed by a third research team member (AT) to ensure the reliability of the transcriptions. Whenever necessary, a member of the research staff reconciled any differences. Interviews followed a semi-structured guide with prompts related to parents’ perception of physical activity, child activity levels and examples, parent and child barriers and opportunities for physical activity, and program-specific questions. Sample questions and prompts included, “List all the ways you can think of that your child is physically active”, “What has helped him/her be active?”, and “What things have gotten in your child’s way when trying to participate in physical activity?” All questions were written by two researchers. Two experts in qualitative inquiry vetted the questions. Multiple rounds of feedback from the experts to the research team occurred to reduce bias. Interview time ranged from 13 to 43 min, with an average time of 23 min.

### 2.4. Analysis

NVivo v.14 software was used to analyze the transcripts. An inductive, naturalistic evaluation approach to qualitative analysis guided this study, following the six recursive phases of thematic analysis [51]. Two researchers who were not involved in the interview process independently familiarized themselves with all the qualitative data through an initial reading of all the transcripts and noting their initial observations. Initial open coding uncovered multiple broad themes related to facilitators and barriers to physical activity for preschool children. Next, the two researchers came together to review the initial themes and sought consensus. Broad themes were only retained if they had been initially coded by each member of the team. Refining themes and generating operational definitions occurred over several meetings and utilized a constant comparative method, requiring consensus before moving to the next stage of analysis. When conflict between researchers’ interpretation of codes or themes arose, the value systems represented were discussed, recommendations were negotiated, and a third researcher was consulted when necessary. The third researcher familiarized themself with the transcripts and helped the team reach a consensus on theme definitions and parameters. The process of independent coding followed by comparison helped to ensure intercoder reliability [52]. The iterative process of peer discussion and debriefing assured trustworthiness. After extensive reflection and deliberation, the recursive process resulted in a final report of agreed-upon themes and examples to support each. The researchers collaboratively identified four major facilitators, four major barriers, and an overarching theme of parental support for childhood physical activity.

## 3. Results

The overarching theme of caregiver encouragement was clear throughout the interviews. This sample of caregivers valued physical activity and encouraged their children to be active, thus explaining the notable outweighing of facilitators over barriers, as demonstrated in Table 2 and Table 3. This was reflected by every caregiver who was interviewed. For example, one caregiver commented:

“*I’ve always seen physical activity as important for your health and just to stay involved and active instead of just sitting around all the time or, you know, I’ve always had my kids active in different activities that involve physical activity and try to, you know, show them the importance of it as well*”.

P2

This sentiment of valuing physical activity and the desire to pass that value on to their children was expressed simply and matter-of-factly by others who stated,

“*I’d rather them be active than inactive*”,

P4

and

“*It’s very important to us, you know, we like to be physically active*”.

P8

All the caregivers represented in these interviews played a meaningful role in encouraging their children to be active at home. Even when unable to articulate specific reasons why, as represented in the general comments above, they clearly recognized that physical activity was valuable and important. 

### 3.1. Facilitators

#### 3.1.1. Access to Resources

The most common facilitator to—physical activity was access to resources, which included environmental and structural resources, knowledge-based resources, and educational opportunities, as well as equipment. Physical resources mentioned by participants included environmental factors such as access to parks, pools, playgrounds, and places to ride bikes, and community factors such as the ability to be involved in youth sports programs. Participants also considered the structure of their physical environment to be an asset. For example, quotes that exemplify a sentiment shared by many were:

“*They will go outside ‘cause we live on three acres of land. They got so much free range far away from the roads*”.

P9

“*He’s outside practicing tee ball, running around, fishing, helping us pick up outside*”.

P11

A separate, yet related subtheme was access to equipment. Various types of equipment, including bicycles, balls, bats, and other sports equipment, were mentioned a total of 25 times by 7 different participants. Having equipment, and specifically the equipment provided by the SKIPping with PALS program, which included a bag filled with poly spots, bean bags, hula hoops, a plastic bat and racquet, and cue cards and photo cards that described the skills and sample activities, allowed the children to engage in physical activity and practice the fundamental skills they had been learning in the program. For example, caregivers noted the following:

“*At home we are doing more because we have that bag of stuff. It was imperative that something like that come into the home to remind not just the kids that they can go do stuff, but to remind the parents of what we used to do*”.

P1

“*And again, providing the materials and resources because I mean otherwise, a lot of our parents would not go out and buy those things*”.

P2

Also categorized as a facilitating resource was access to knowledge-based sources such as books, informational materials, and health professionals to consult with. These intangible, knowledge-generating resources were mentioned by all 11 caregivers. While these caregivers already valued physical activity, most mentioned learning something new from the SKIPping with PALS program that further reinforced either their desire or ability to support their children in being active. Those who learned new things that motivated them to think differently about their kids being active said things such as:

“*I didn’t realize that there was such a connection between being physically active and being able to achieve milestones that reflected school achievements, writing and reading, so that was pretty cool*”.

P8

“*This year has been an eye-opener as to how much my child doesn’t know about certain activities or not capable of doing that. I know I was capable of doing, so I’m astounded that I never caught on that he didn’t have those capabilities*”.

P1

“*A lot of people just say, ‘they’ll figure it out, just let them do it’…but I was surprised that there is learning about how…your children does the hand-eye coordination to proper movements in the proper way to do the exercises*”.

P5

Those who learned new things that helped them better engage their kids in physical activity said things such as:

“*Having the knowledge from the sessions to be able to implement some of those things and not have to try to come up with activities, you know off the top of my head, or having to go out and find things to do—it’s already provided*”.

P2

“*It’s given me other activities to do, gives me something different to do outside*”.

P7

One of the educational resources provided by the SKIPping with PALS program was informational cards with simple activity ideas that they referred to as cue cards. Almost half (*n* = 5) of the caregivers specifically mentioned this resource as especially helpful.

#### 3.1.2. Benefits to Children

All of the caregivers in this study recognized that physical activity was beneficial for children and that sense seemed to be a driving factor for encouraging physical activity in the home environment. The specific benefits that they mentioned include academic, health and development, reducing sedentary tendencies, and expending energy.

One participant exemplified the idea of academic benefits of physical activity when she said:

“*If he’s been outside running around he is more apt to sit down and want to look at the book and want to look at the actual words. He’s not as hyped up as he would normally be so then he could actually focus on the book and mathematics and stuff*”.

P8

References to health and developmental benefits were generally more vague. For example,

“*I think it’s very important just so you aren’t like sedentary for health reasons and also it just makes you feel better*”.

P10

“*I think that for kids, it’s a developmental need. I think that without it kids do not grow or function the way they should*”.

P1

Some parents mentioned the benefit of physical activity in relation to reducing technology use, such as:

“*It is important for kids to be physically active. I think that they shouldn’t, you know, always have the screen time as their primary mode of play*”.

P10

Another explained why she encourages her child to be active by describing her child as:

“*a firecracker and or a rocket, they’re just constant and they need something or else it’s going to build up*”.

P1

The benefits mentioned by caregivers were numerous and varied, but clearly a driving force in their home routine.

#### 3.1.3. Social Factors

Social factors were a significant driving force for physical activity in this sample, mentioned by 9 of the 11 caregivers. Central among these comments was the importance of fostering parent–child connections. Caregivers said things such as:

“*It initiates family time*”.

P1

“*It’s really given us a bonding time*”.

P11

Many caregivers talked about the importance of being active with their children as a way to invest in and spend time with them. For example:

“*The boys enjoy it. I mean they enjoy my interaction with them and kind of making them feel special about me taking time out of my day just for them*”.

P3

“*It’s always fun to be involved with what your kids do and show them that, you know, you care about them, that you’re aware with what goes on in their lives*”.

P5

Also amongst social factors was the opportunity to engage with other children, particularly those who serve as role models. This is exemplified by comments such as:

“*Him seeing us be active in things helps motivate him. Having older siblings that are active*”.

P2

“*Being around me and his older brother and cousin, just seeing other kids doing these things, then he wants to do them*”.

P5

#### 3.1.4. Child Personality and Enjoyment

Finally, enjoyment of -physical activity and an active personality were driving factors in caregivers’ motivation to support their children’s physical activity behaviors. Participants pointed out that it is easier to encourage children to be active who generally enjoy being active. The words “he/she just loves to be active” or “he/she enjoys it” were the exact words of five caregivers, and four specifically mentioned a love of being outdoors and connected that to being active. Caregivers who perceived their children to be especially energetic emphasized the inherent value of physical activity in helping to meet the personality demands of their children, such as:

“*He’s full speed ahead, he’s very athletic*”.

P3

“*She’s always running somewhere, she never slows down*”.

P4

It is notable that the least common facilitator (personality and enjoyment, *n* = 9) was mentioned far more often than the most common barrier (weather, *n* = 4). However, even within this sample that strongly valued physical activity, and where facilitators of physical activity far outweighed barriers, caregivers did articulate multiple barriers that can help elucidate what makes it easier or harder for them to follow through on their internal commitment to helping their children be active throughout the day. 

### 3.2. Barriers

#### 3.2.1. Weather

Weather was the most common barrier mentioned by four caregivers. Most participants described their children’s activity as occurring outside, thus inclement weather presented a challenge related to their perceptions about the types of activities that could be performed inside. Caregivers remarked that being physically active depended on the weather and during unbearable heat or rain, the children

“*…can’t go outside, so they’re stuck inside the house*”.

P5

inferring that they were inactive when inside.

#### 3.2.2. Caregiver Constraints

Caregiver constraints, including time, health, and childcare, were a major barrier, tied to other secondary barriers such as safety, technology, school, and resources. Most caregivers referred to the challenge of juggling work schedules and time with their children. Comments included:

“*I would say time constraints, like I work 12-h shifts and so does her dad so her grandma tries to, you know, to make sure she can get outside but she has you know, younger children to take care of, too*”.

P10

“*Just because of the longer hours we’re having to do right now. I’m not able to get out of the area to be home and do more physical activity with him*”.

P11

#### 3.2.3. Child’s Disability

Child’s disability was mentioned as a barrier by three participating caregivers. Children of caregivers represented in this sample had documented disabilities including hearing impairment, chronic illness, and learning disabilities. For example, when describing challenges with helping their child to be physically active, one caregiver said:

“*I think a lot of it has to do with him having a learning disability…when he gets frustrated his focus goes out the window*”.

P9

This challenge with attention and managing frustration seemed to be a barrier when participating in structured physical activities the child had participated in in the past. However, the same caregiver noted that the SKIPping with PALS program helped to overcome that barrier. They said:

“*But you know with going to PALS and the teachers at school and everything they help him because they give him a schedule and that actually helps him*”.

P9

#### 3.2.4. Technology

Technology was mentioned as a barrier competing for the child’s time and attention. This was related to parental constraints in the sense that children would resort to using technology when their caregivers were not directly engaging them in an activity. Caregivers said things such as:

“*They’re in the confines where it’s a TV, a tablet, maybe playing some games but not nearly as physical as we would want to be. With my husband and I home, they get a little more tablet time than they should*”.

P1

“*I think it’s a little different just because of the new technology and everything kind of distracts*”.

P2

## 4. Discussion

The purpose of this study was to examine the facilitators and barriers to childhood physical activity as perceived by parents living in low socioeconomic status and rural areas of the USA. Overall, facilitators were mentioned by participants far more than barriers in both frequency and impact. Despite the social determinants of health that could create structural barriers for most of these families (e.g., low income and rural geography), these were caregivers who saw past those and encouraged their children to be active. The most common facilitator to physical activity was access to resources, which is notable given that these families all live below the poverty line. While their financial resources were, in general, limited, they did not find this to be a barrier to childhood physical activity and, in fact, often mentioned free community resources such as parks, playgrounds, and recreation programs as facilitators. Notably, while rural geography is often considered a barrier to physical activity [30,31], caregivers in this sample saw the rural setting as an asset, not a barrier. They talked about the value of their children having space to run and play, woods to explore, and freedom to be active outside in a safe setting, which is consistent with the findings from Hinkley et al. [53].

While the SKIPing with PALS program, overall, was an important educational resource, the bag of equipment provided to all participants seemed to be especially impactful towards enabling opportunities for the children to engage in types of activities with which they otherwise would not be able. Furthermore, the information that caregivers learned through the SKIPping with PALS program seemed to reinforce their desire to keep their children active, demonstrating the value of educational programs that engage parents in understanding the nuances of gross motor development, as well as practical skill building to prepare parents to engage in active ways with their children. These findings are consistent with the recommendations from the systematic review conducted by Venetsanou and Kambas [54], as well as the cross-sectional data investigated by Barnett et al. [55] where parents were considered a modifiable environmental factor, supporting movement behavior and motor competence. Moreover, the perceived value of the informational cards included in the equipment bag emphasizes the importance of access to quick, tangible knowledge-based resources that are not technology-based, particularly for families who live in rural settings where internet access is less available or reliable.

In contrast to the literature which suggests that families living in rural settings lack access to social supports [30,31], the caregivers in this program identified social supports as a major facilitator of their child’s physical activity. The family was the most referred to social factor and included parents, grandparents, siblings, and cousins. Many caregivers talked about the importance of physical activity as a bonding time, which highlights the value of activities that are both fun and active. The parental desire to spend time with their children doing something that they all enjoyed was a driving factor for many of these families. The social aspect, which includes role modeling and parent–child connection, is supported by previous research that has also shown that social support and parental role modeling, specifically, are associated with higher levels of physical activity among young children [56,57].

Notably, some of the social factors referenced by the caregivers in this sample were more circumstantial (e.g., number of children in a family and living proximity to other children or family) and/or attitudinal (e.g., parent desire to participate in physical activity), which are difficult to modify through traditional educational programs. However, social factors are important components of physical activity. This study further emphasizes the value of facilitating social components of physical activity. Thus, including parents and caregivers in physical activity interventions for young children, as well as older siblings and other close family members who can serve as a source of social support, could be of the utmost importance. 

Although facilitators far outweighed any barriers mentioned by participants, the following four themes emerged: inclement weather, caregiver constraints, child’s disability, and competing technology. Weather has been cited as a barrier in the previous literature and points to the need to incorporate indoor activity options as part of early childhood physical activity programs [53,56]. SKIPping with PALS did this by incorporating the use of household items to practice motor skills with limited space and resources, which may explain why weather was not mentioned as a barrier by most of the participants.

Caregiver constraints, while not unique to these families, are putative barriers to increasing physical activity among young children [53,56]. The caregivers in this sample who were without time or health constraints were better able to overcome secondary barriers such as technology use and geographic constraints. This resilience indicates that families with known significant challenges, such as two working parents, need additional support and resources to help them work within their legitimate barriers on time and energy. Unsurprisingly, the competing appeal of technology was a stark barrier for families with caregiver constraints. The challenge of children’s use of technology is not unique to this study. This barrier is consistent with a larger societal trend that parents are facing today and is one that is not easily solved [58]. 

Child’s disability, which included hearing impairment, chronic illness, and learning disabilities, was perceived as a barrier by caregivers in this study. While they represented a small proportion of children in the SKIPping with PALS program, the frequent mention of the disability is notable. Children with disabilities face additional challenges and limited access to structured physical activity, especially in rural settings [59]. However, when appropriate structures and supports are provided, children with disabilities can thrive and benefit from a physical activity program, as noted by one of the participants in this study. Instructional and behavioral supports such as schedules, cue cards, and prompting, which were used in the PALS program, are important components that facilitate positive and successful learning experiences for all children with a variety of abilities and disabilities [1]. Thus, building these types of supports into future programs has the potential to mitigate the perception of disability as a barrier and to allow all children, regardless of ability, to experience success and yield the benefits of physical activity. 

### Limitations and Future Research

Although many interesting and important findings resulted from this novel inquiry, this study is not without limitations. Readers should interpret all findings with caution. Given that the purpose of qualitative research is to derive meaning from rich and in-depth inquiry from a smaller group of participants, findings may not be generalizable, inferentially, to a larger population, as that was not the intended purpose of this paradigm. Specifically, these data should be interpreted under the auspices that our population was from rural, Southern United States and a low socioeconomic environment. Inference to other locations, although not the purpose of qualitative inquiry, should not be made. However, these rich data can be used to support and inform future inquiries seeking to support/promote physical activity behaviors in residents of areas historically at-risk for adverse health effects resulting from high bouts of sedentary behavior. Although the participants likely agreed to participate in the interviews for this study based upon their values and positive attitudes towards physical activity and education, their insights can glean valuable contributions that inform content within future programs to promote physical activity behaviors in rural communities. 

Future research projects should focus on the influence of intervention programs that target the facilitators of physical activity, as identified by these participants, to prevent or remediate excessive sedentary behavior by promoting health-enhancing levels of physical activity. Understanding what motivates people only strengthens future endeavors. 

## 5. Implications for Practice and Conclusions

Decisions to participate in physical activity behaviors are typically the result of a mix of the following three factors: task, environment, and person. Understanding the individual and their physical, psychological, and cognitive characteristics can inform options that are sustainable and viable within any given environment. The children represented in this study did not regularly possess the agency to be physically active without parental support as they were 3-6 years of age. As a result, caregivers are either a primary environmental barrier or facilitator, influencing the physical activity behaviors of young children. Caregivers who value PA have the capacity and desire to encourage their children to be active and, with minimal levels of support, they can establish a strong foundation for the development of lifetime physical activity skills and dispositions. Emphasizing the crucial role of caregiver education is essential in empowering them to act as facilitators for their children, particularly by providing them with comprehensive guidance on the significance and practicalities of physical activity behaviors and goal-directed movement skills. Community-based interventions and educational programs should provide caregivers a wide range of physical activity ideas that can help children discover various activities that they enjoy and strategies for how to build social support around physical activity, especially within the family unit. Caregiver education can combat the existing disparities often facing those from rural and impoverished settings.

Paramount for those from impoverished settings is providing structured physical activity programming where the families can learn, move, and come to value being active in a manner that results in success and goal attainment. Providing options for indoor activities can enhance outcomes where weather can be a factor. The impact of structured physical activity opportunities can be most fruitful long-term when caregivers and possibly teachers (if available) can serve as role models. The value-added outcome of teaching caregivers and teachers to value and understand physical activity opportunities that are goal-directed is that both children and adults now reap health-related benefits of movement opportunities, holistically (e.g., psychosocial, physical, and cognitive). Overall, residing in a rural setting can be an asset (as opposed to a barrier) when the spaces are utilized efficiently and families are supported through community or school-based programming that provides the resources (e.g., equipment bags and low-tech educational resources) to those experiencing poverty. These recommendations are in line with the Ecological Model purported by Sallis and colleagues in recognition of the requisite domains to promote active living, specifically social support structures that begin with the family [29].

Programs such as SKIPping with PALS play a pivotal role in fostering positive developmental trajectories for the health-related quality of life within the community, starting in early childhood. This is accomplished through the mitigation of disparities prevalent in rural and low-income areas by eliminating task and environmental constraints and providing tailored support to individuals in a manner that aligns with their developmental needs.

## Figures and Tables

**Table 1:** Summary of participant demographic information.

Caregiver Role	Caregiver Sex	Caregiver Race	Child Sex	Child Race	Child Age (Months)
Mother	Female	White	Male	White	62
Mother	Female	White	Male	White	67
Mother	Female	White	Male	White	56
Grandmother	Female	Black	Female	Black	53
Father	Male	Black	Male	Black	52
Mother	Female	White	Female	White	60
Mother	Female	Black	Female	Black	54
Mother	Female	White	Male	White	43
Mother	Female	White	Male	White	65
Mother	Female	White	Female	White	65
Mother	Female	White	Female	White	67

**Table 2:** Facilitators to Early Childhood Physical Activity.

Facilitators	Number of People Who Mentioned the Theme (n)	Number of Unique Mentions of the Theme (n)
Access to resources	11	88
Environmental and structural	11	26
Knowledge and education	11	42
Equipment	8	25
Benefits to children	11	40
General	6	13
Academic	6	7
Health and development	6	10
Minimizing sedentary tendencies	4	6
Energy expenditure	3	4
Social factors	9	37
Fostering caregiver–child connection	8	17
Role modeling	7	11
Engagement with other children	5	9
Child personality and enjoyment	9	27

*N* = 11. Category totals do not equal the sum of subthemes because the complexity of some statements caused them to be coded into multiple areas.

**Table 3:** Barriers to early childhood physical activity.

Barriers	Number of People Who Mentioned the Theme (n)	Number of Unique Mentions of the Theme (n)
Weather	4	7
Caretaker constraints	4	5
Technology	3	5
Child disability	3	5

*N* = 11.

## Data Availability

The data presented in this study are available on request from the corresponding author. The data are not publicly available due to specific ethical and privacy considerations.
